# Green Synthesis of New Category of Pyrano[3,2-c]Chromene-Diones Catalyzed by Nanocomposite as Fe3O4@SiO2-Propyl Covalented Dapsone-Copper Complex

**DOI:** 10.3389/fchem.2021.720555

**Published:** 2021-09-01

**Authors:** Leila Zare Fekri

**Affiliations:** Department of Chemistry, Payame Noor University, Tehran, Iran

**Keywords:** dapsone-cu, silica-coated Fe3O4, silica, pyranochromenes, nanoorganometallic chemistry

## Abstract

Nanomagnetic dapsone-Cu supported on the silica-coated Fe_3_O_4_ (Fe_3_O_4_@SiO_2_-pr@dapsone-Cu) nanocomposite was synthesized and characterized by Fourier transform infrared (FT-IR), energy-dispersive X-ray (EDX), X-ray diffraction (XRD), field emission scanning electron microscope (FE-SEM), transmission electron microscopy (TEM), zeta potential, vibrating sample magnetometer (VSM), and thermogravimetric analysis (TGA). This newly synthesized nanocomposite was chosen to act as a green, efficient, and recyclable Lewis acid for the multicomponent synthesis of new derivatives of pyrano[3,2-c]chromene-diones through the reaction of aromatic aldehydes, indandione, and 4-hydroxycoumarin in water. All of the synthesized compounds are new and are recognized by FT-IR, NMR, and elemental analysis; this avenue is new and has advantages such as short reaction times, high productivity, economical synthesis, and use of green solvent, H_2_O, as a medium. The catalyst is magnetically recoverable and can be used after six runs without a decrease in the efficiency.

## Introduction

Multicomponent reactions (MCRs) are useful avenue for the synthesis of organic compounds. This reaction is a combination of at least three components in a one-pot domino (Li and Chan, 1997; Grieco, 1998; Dömling and Uzi, 2000; Dömling, 2002; Hosseini-Zare et al., 2012). MCRs have benefits such as higher atom economy and selectivity and production of complex molecules with low by-products. Nowadays, MCRs have attracted a lot of interest in organic transformation (Bienaymé et al., 2000; Kandhasamy and Gnanasambandam, 2009; Müller 2014).

Pyranochromenes have various biological activities such as antifungal (Ouf et al., 2014), antibacterial (Zhang et al., 2012), antihyperglycaemic and antidyslipidemic (Kumar et al., 2009), anticancer (Lee et al., 2009), cytotoxic (Magiatis et al., 1998), anti-HIV (Kongkathip et al., 2005), anti-HBV (Su et al., 2009), antiviral (Barnard et al., 2002), antiproliferative (Mao et al., 2019), anti-inflammatory (Symeonidis et al., 2009), antinociceptive (Lima et al., 2006), and antituberculosis (Xu et al., 2006). They are also synthetic intermediates for alkaloids, drug candidates, and clinical pharmaceuticals (Williams and Cox, 2003).

Magnetic nanoparticles (MNPs) as catalysts are interesting materials because of their high surface area, unique magnetic properties, and high catalytic activity. They were used as magnetic fluids catalysis and magnetic resonance imaging (MRI) data storage and environmental remediation (Rezaee Nezhad et al., 2014; Mokhtary, 2016; Rezayati et al., 2016; Fekri and Maleki, 2017; Abbasi et al., 2017; Fekri et al., 2020; Sardarian et al., 2019; Inaloo et al., 2020a; 2020b; 2020c; 2020d).

To the best of our knowledge, there are no reports on the use of Fe3O4@SiO2-propyl-loaded dapsone-copper as a catalyst for the synthesis of pyrano[3,2-c]chromenes via multicomponent reactions of aldehydes, indandione, and 4-hydroxycoumarin results.

## Experimental

### Material and Method

The X-ray diffraction (XRD), transmission electron microscopy (TEM), scanning electron microscope (SEM), thermogravimetric analysis (TGA), and zeta potential analysis for synthesized MNPs were analyzed on X-PRTPRO (Netherlands) XRD, TEM Jeol model 3,010, Philips XL 30 scanning electron microscope (SEM, Netherlands), Q600 (made in America), and ZEN3600 (England) instrument, respectively. Fourier transform infrared (FT-IR) spectra of organic compounds were carried out by Shimadzu Fourier transform infrared spectrophotometer (FT-IR-470, Japan). 1H and 13C NMR spectra were determined using a Bruker DRX Avance instrument at 500 or 300 and 125 or 75 MHz.

### General Procedure for Preparation of Fe_3_O_4_

Nanoparticles Fe_3_O_4_ were synthesized by Zare Fekri (Fekri and Maleki, 2017; Fekri, 2020).

### Synthesis of Fe3O4@SiO2-Cl

Fe_3_O_4_@SiO_2_ NPs were prepared by Zare Fekri (Nikpassand et al., 2017; Fekri et al., 2020; Fekri and Zeinali, 2020).

### Synthesis of Fe_3_O_4_@SiO_2_@dapsone

500 mg Fe_3_O_4_@SiO_2_-Cl MNPs in 50 ml distilled water were irradiated under ultrasound for 30 min. Then, dapsone 0.5 g was added. The mixture was refluxed at 110°C for 14 h. The Fe_3_O_4_@SiO_2_@dapsone was filtered in the presence of an enormous magnet and washed with chloroform several times and dried at 80°C for 4 h.

### Synthesis of Fe_3_O_4_@SiO_2_@dapsone-Cu

500 mg Fe_3_O_4_@SiO_2_@dapsone MNPs in 50 ml EtOH-H_2_O (1:1) were irradiated under ultrasonic bath for 30 min. Then, 20 ml aqueous solution of copper chloride (I) (0.1 g; 0.001 mol) was added to the Fe_3_O_4_@SiO_2_@dapsone and stirred for 48 h. The Fe_3_O_4_@SiO_2_@dapsone-Cu MNPs were filtered in the presence of a magnetic bar and washed using ethanol and water subsequently, to separate the nanoparticles.

### General Procedure for the Synthesis of Pyrano[3,2-c]chromene-Dione

A mixture of aldehyde (1.0 mmol), indan-1,3-dione (2.0 mmol), 4-hydroxycoumarin (1 mmol), and 0.05 g Fe_3_O_4_@SiO_2_@dapsone-Cu MNPs was stirred at room temperature in 10 ml distilled water for the required reaction time as indicated by TLC (TLC silica gel 60 F250, ethyl acetate : n-hexane 1 : 4). After completion of the reaction, the resulting mixture was filtered in the presence of an efficient magnetic bar to separate the catalyst. The catalyst was washed with 10 ml ethanol and reused. The crude products were collected and dried.

### Characterization Data

7-(4-Nitrophenyl)-6H-indeno [2',1':5,6]pyrano [3,2-c]chromene-6,8(7H)-dione 4a: m. p. 203–205°C, FT-IR (KBr, cm^−1^): 1739 (C=O str), 1701 (C=O str), 1,561 (Asymmetric NO_2_ str or aromatic C=C str), 1,510, 1,456 (aromatic C=C str), 1,348 (symmetric NO_2_ str), 1,257 (C-O str), 1,099.^1^H NMR (DMSO-d_6_, 400 MHz): δ_H_ 5.31 (s, 1H, chiral C-H), 6.84–6.88 (m, 1H, Ar), 7.00–7.04 (m, 1H, Ar), 7.18 (d, J = 8.0Hz, 1H, Ar), 7.23 (d, J = 2.4Hz, 1H, Ar), 7.31 (d, J = 8.0Hz, 1H, Ar), 7.35 (s, 1H, Ar), 7.55 (d, J = 8.0Hz, 2H, Ar), 7.85–7.95 (m, 2H, Ar), 8.06–8.14 (m, 1H, Ar) ppm. ^13^C NMR (DMSO-d_6_, 100 MHz): δC 57.50 (chiral carbon), 111.60 (Ar), 113.45 (Ar), 117.89 (Ar), 121.98 (two peaks, Ar), 123.56 (Ar), 123.98 (Ar), 125.01 (Ar), 126.23 (Ar), 127.09 (Ar), 131.56 (Ar), 131.87 (Ar), 132.33 (Ar), 137.89 (Ar), 133.98 (Ar), 139.09 (two peaks, Ar), 141.23 (Ar), 145.81 (Ar), 149.87 (Ar), 198.76 (C=O), 201.17 (C=O)ppm. Anal. Calcd. for C_25_H_13_NO_6_: C, 70.92; H, 3.09; N, 3.31. Found: C, 70.93; H, 3.05; N, 3.32.

7-(3-Nitrophenyl)-6H-indeno [2',1':5,6]pyrano [3,2-c]chromene-6,8(7H)-dione 4b: m. p. 176–177°C, FT-IR (KBr, cm^−1^): 1736 (C=O str), 1700 (C=O str), 1,660, 1,580 (asymmetric NO_2_ or aromatic C=C str), 1,430 (aromatic C=C str), 1,330 (symmetric NO_2_ str), 1,275 (C-O str). ^1^H NMR (DMSO-d_6_, 400 MHz): δ_H_ 5.33 (s, 1H, chiral C-H)), 6.85 (t, J = 7.2Hz, 1H, Ar), 7.00 (t, J = 7.2Hz, 1H, Ar), 7.22 (d, J = 8.4Hz, 2H, Ar), 7.31 (d, J = 8.4Hz, 1H, Ar), 7.35 (d, J = 2.0Hz, 1H, Ar), 7.52 (t, J = 8.0Hz, 1H, Ar), 7.78 (d, J = 8.0Hz, 1H, Ar), 7.85–7.94 (m, 2H, Ar), 8.00–8.02 (m, 1H, Ar), 8.18 (d, J = 2.0Hz, 1H, Ar) ppm. ^13^C NMR (DMSO-d_6_, 100 MHz): δ_C_ 58.30 (chiral C), 112.00 (two peaks, Ar), 113.54 (Ar), 114.00 (Ar), 118.96 (Ar), 119.18 (Ar), 121.80 (Ar), 122.03 (Ar), 123.29 (Ar), 123.33 (Ar), 124.75 (Ar), 126.61 (Ar), 129.98 (two peaks, Ar), 135.84 (Ar), 136.36 (Ar), 136.66 (Ar), 142.40 (Ar), 142.44 (Ar), 144.60 (Ar), 147.91 (Ar), 198.90 (C=O), 200.16 (C=O) ppm. Anal. Calcd. for C_25_H_13_NO_6_: C, 70.92; H, 3.09; N, 3.31. Found: C, 70.95; H, 3.07; N, 3.36.

7-(4-Bromophenyl)-6H-indeno [2',1':5,6]pyrano [3,2-c]chromene-6,8(7H)-dione 4c: m. p. 276–278°C, FT-IR (KBr, cm^−1^): 1735 (C=O str), 1700 (C=O str), 1,650, 1,457 (aromatic C=C str), 1,215 (C-O str). ^1^H NMR (DMSO-d_6_, 400 MHz): δ_H_ 5.13 (s, 1H, chiral C-H), 6.85 (t, J = 7.2Hz, 1H, Ar), 7.01 (t, J = 7.2Hz, 1H, Ar), 7.13 (d, J = 8.0Hz, 1H, Ar), 7.17 (d, J = 8.4Hz, 2H, Ar), 7.31 (dd, J = 3.2Hz, J = 5.6Hz, 2H, Ar), 7.35 (dd, J = 1.6Hz, J = 6.4Hz, 2H, Ar), 7.84–7.93 (m, 2H, Ar) ppm. ^13^C NMR (DMSO-d_6_, 100 MHz): δC 58.48 (chiral C), 111.91 (Ar), 114.32 (Ar), 118.95 (Ar), 119.00 (Ar), 119.99 (Ar), 121.60 (Ar), 123.25 (Ar), 124.76 (Ar), 126.71 (two peaks, Ar), 131.06 (Ar), 131.21 (Ar), 131.27 (Ar), 136.44 (Ar), 136.54 (Ar), 136.58 (Ar), 141.35 (Ar), 142.38 (Ar), 142.61 (Ar), 144.32 (Ar), 197.56 (C=O), 200.26 (C=O) ppm. Anal. Calcd. for C_25_H_13_BrO_4_: C, 65.66; H, 2.87. Found: C, 65.65; H, 3.07; N, 2.86.

7-(3-Hydroxyphenyl)-6H-indeno [2',1':5,6]pyrano [3,2-c]chromene-6,8(7H)-dione 4d: m. p. 289–290°C, FT-IR (KBr, cm^−1^): 1737 (C=O str), 1705 (C=O str), 1,650, 1,608 (aromatic C=C str), 1,538, 1,224 (C-O str). ^1^H NMR (DMSO-d6, 400 MHz): δ_H_ 5.04 (s, 1H, chiral C-H), 6.41–6.44 (m, 1H, Ar), 6.57 (t, J = 7.8Hz, 1H, Ar), 6.92 (d, J = 8.7Hz, 1H, Ar), 6.84 (t, J = 8.5Hz, 1H, Ar), 6.90 (t, J = 8.5Hz, 1H, Ar), 7.00–7.04 (m, 1H, Ar), 7.14 (d, J = 8.5Hz, 1H, Ar), 7.32 (d, J = 7.8Hz, 1H, Ar), 7.41 (d, J = 4.3Hz, 1H, Ar), 7.83–7.92 (m, 3H, Ar), 9.13 (s, 1H, OH) ppm. ^13^C NMR (DMSO-d6, 100 MHz): δ_C_ 58.71 (chiral C), 111.83 (Ar), 113.91 (two peaks, Ar), 115.11 (Ar), 116.07 (Ar), 118.86 (two peaks, Ar), 118.99 (Ar), 119.63 (Ar), 121.48 (Ar), 123.15 (two peaks, Ar), 124.78 (Ar), 126.94 (Ar), 129.23 (Ar), 136.36 (two peaks, Ar), 136.43 (Ar), 142.45 (Ar), 142.85 (two peaks, Ar), 143.06 (Ar), 157.23 (Ar), 197.83 (C=O), 200.51 (C=O) ppm. Anal. Calcd. for C_25_H_14_O_5_: C, 76.14; H, 3.58. Found: C, 76.11; H, 3.07.

7-(4-(Methylthio)phenyl)-6H-indeno [2',1':5,6]pyrano [3,2-c]chromene-6,8(7H)-dione 4e: m. p. 269–270°C, FT-IR (KBr, cm^−1^): 2,921 (aliphatic C-H str), 1735 (C=O str), 1701 (C=O str), 1,593 (aromatic C = c str), 1,488 (aromatic C=C str), 1,423, 1,344, 1,261 (C-O str). ^1^H NMR (DMSO-d_6_, 400 MHz): δ_H_ 2.43 (s, 3H, SCH_3_), 5.10 (s, 1H, chiral C-H), 6.84 (t, J = 7.8Hz, 1H, Ar), 7.01 (d, J = 8.5Hz, 3H, Ar), 7.13 (d, J = 8.5Hz, 3H, Ar), 7.31 (d, J = 3.2Hz, 1H, Ar), 7.36 (s, 1H, Ar), 7.86–7.92 (m, 2H, Ar) ppm. ^13^C NMR (DMSO-d6, 100 MHz): δ_C_ 51.63 (SCH_3_), 58.75 (chiral C), 112.98 (Ar), 113.93 (two peaks, Ar), 114.01 (Ar), 115.08 (Ar), 116.81 (Ar), 117.23 (Ar), 119.53 (Ar), 120.56 (Ar), 122.05 (two peaks, Ar), 124.89 (Ar), 125.34 (Ar), 125.87 (Ar), 134.21 (Ar), 136.42 (Ar), 143.21 (two peaks, Ar), 145.17 (Ar), 158.23 (Ar), 196.23 (C=O), 201.01 (C=O) ppm. Anal. Calcd. for C_26_H_16_O_4_S: C, 73.57; H, 3.80. Found: C, 73.51; H, 3.87.

7-(Pyridin-3-yl)-6H-indeno [2',1':5,6]pyrano [3,2-c]chromene-6,8(7H)-dione 4f: m. p. >300°C, FT-IR (KBr, cm^−1^): 3,034 (aromatic C-H str), 2,910 (aliphatic C-H str), 1743 (C=O str), 1703 (C=O str), 1,565 (aromatic C=C str), 1,425 (aromatic C=C str), 1,265 (C-O str), 1,168.^1^H NMR (DMSO-d_6_, 400 MHz): δ_H_ 5.21 (s, 1H, chiral C-H), 6.86 (t, J = 7.8Hz, 1H, Ar), 7.02 (t, J = 7.5Hz, 1H, Ar), 7.18–7.22 (m, 2H, Ar), 7.30 (t, J = 8.5Hz, 3H, Ar), 7.65 (d, J = 8.6Hz, 1H, Ar), 7.87–7.94 (m, 3H, Ar), 8.31 (d, J = 7.8Hz, 1H, Ar) ppm. ^13^C NMR (DMSO-d_6_, 100 MHz): δ_C_ 56.78 (chiral C), 113.09 (two peaks, Ar), 114.56 (Ar), 116.12 (Ar), 116.91 (Ar), 116.99 (Ar), 118.13 (Ar), 118.76 (Ar), 121.43 (two peaks, Ar), 122.16 (two peaks, Ar), 123.54 (Ar), 125.57 (Ar), 126.47 (Ar), 128.46 (Ar), 134.09 (Ar), 138.11 (two peaks, Ar), 147.87 (Ar), 155.08 (Ar), 197.34 (C=O), 200.07 (C=O) ppm. Anal. Calcd. for C_24_H_13_NO_4_: C, 75.98; H, 3.45; N, 3.69. Found: C, 75.93; H, 3.43, N, 3.65.

7-Phenyl-6H-indeno [2',1':5,6]pyrano [3,2-c]chromene-6,8(7H)-dione 4 g: m. p. 203–205 °C, FT-IR (KBr, cm^−1^): 3,098 (aromatic C-H str), 1735 (C=O str), 1702 (C=O str), 1,603 (aromatic C=C str), 1,408, 1,365, 1,323, 1,211 (C-O str). ^1^H NMR (DMSO-d_6_, 400 MHz): δ_H_ 5.10 (s, 1H, chiral C-H), 6.35–6.56 (m, 3H, Ar), 6.68 (d, J = 7.8Hz, 2H, Ar), 6.86 (s, 1H, Ar), 6.98–7.11 (m, 2H, Ar), 7.28 (d, J = 6.8Hz, 3H, Ar), 7.34 (d, J = 8.2Hz, 2H, Ar) ppm. ^13^C NMR (DMSO-d_6_, 100 MHz): δ_C_ 58.52 (chiral C), 114.23 (Ar), 114.38 (Ar), 116.01 (Ar), 116.98 (Ar), 117.45 (Ar), 117.67 (Ar), 117.89 (Ar), 121.32 (Ar), 121.67 (Ar), 123.55 (Ar), 124.78 (Ar), 126.98 (Ar), 127.46 (Ar), 129.00 (Ar), 132.65 (two peaks, Ar), 136.89 (Ar), 138.91 (Ar), 141.24 (Ar), 143.67 (Ar), 196.34 (C=O), 200.32 (C=O) ppm. Anal. Calcd. for C_25_H_14_O_4_: C, 79.36; H, 3.73. Found: C, 79.31; H, 3.77.

7-(*p*-Tolyl)-6H-indeno [2',1':5,6]pyrano [3,2-c]chromene-6,8(7H)-dione 4h: m. p. 234–235°C, FT-IR (KBr, cm^−1^): 1734 (C=O str), 1701 (C=O str), 1,576 (aromatic C=C str), 1,435 (aromatic C=C str), 1,387, 1,234 (C-O str), 1,114.^1^H NMR (DMSO-d_6_, 400 MHz): δ_H_ 2.35 (s, 3H, Ph-CH_3_), 5.31 (s, 1H, chiral C-H), 6.87 (d, J = 7.8Hz, 2H, Ar), 6.87–7.09 (m, 2H, Ar), 7.23 (J = 8.5Hz, 1H, Ar), 7.56 (d, J = 7.8Hz, 2H, Ar), 7.67–7.83 (m, 3H, Ar), 8.01–8.09 (m, 2H, Ar) ppm. ^13^C NMR (DMSO-d_6_, 100 MHz): δ_C_ 34.23 (benzylic carbon), 58.65 (chiral C), 112.67 (Ar), 114.00 (Ar), 115.34 (Ar), 116.46 (Ar), 118.87 (two peaks, Ar), 119.45 (Ar), 121.33 (Ar), 125.67 (Ar), 126.23 (Ar), 128.98 (Ar), 129.00 (two peaks, Ar), 132.46 (Ar), 135.47 (Ar), 135.88 (Ar), 137.98 (Ar), 141.12 (Ar), 143.34 (Ar), 146.54 (Ar), 198.08 (C=O), 200.23 (C=O) ppm. Anal. Calcd. for C_26_H_16_O_4_: C, 79.58; H, 4.11. Found: C, 79.53; H, 4.07.

## Result and Discussion

### Synthesis and Characterization

**SCHEME 1:**
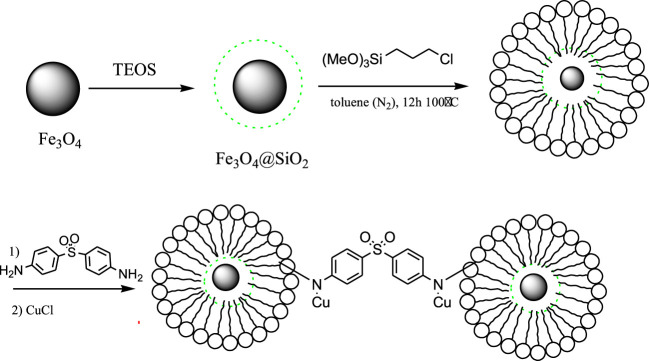
Multistep synthesis of Fe_3_O_4_@SiO_2_-propyl@dapsone-Cu complex.

In order to prepare nanocatalyst, initially, Fe3O4 MNPs were modified with silica and then with chloropropyl silane via chemical bonds to obtain Fe3O4@SiO2-pr. In the next step, Fe3O4@SiO2-propyl was covalented by substitution reaction by dapsone to prepare Fe3O4@SiO2-propyl loaded dapsone. This nanocatalyst was treated with copper chloride to produce Fe3O4@SiO2-propyl@dapsone-Cu (Scheme 1). The structure of the prepared nanocatalyst was studied and fully characterized using FT-IR, energy-dispersive X-ray (EDX), XRD, zeta potential, TEM, and field emission scanning electron microscope (FE-SEM) analysis.

**FIGURE 1:**
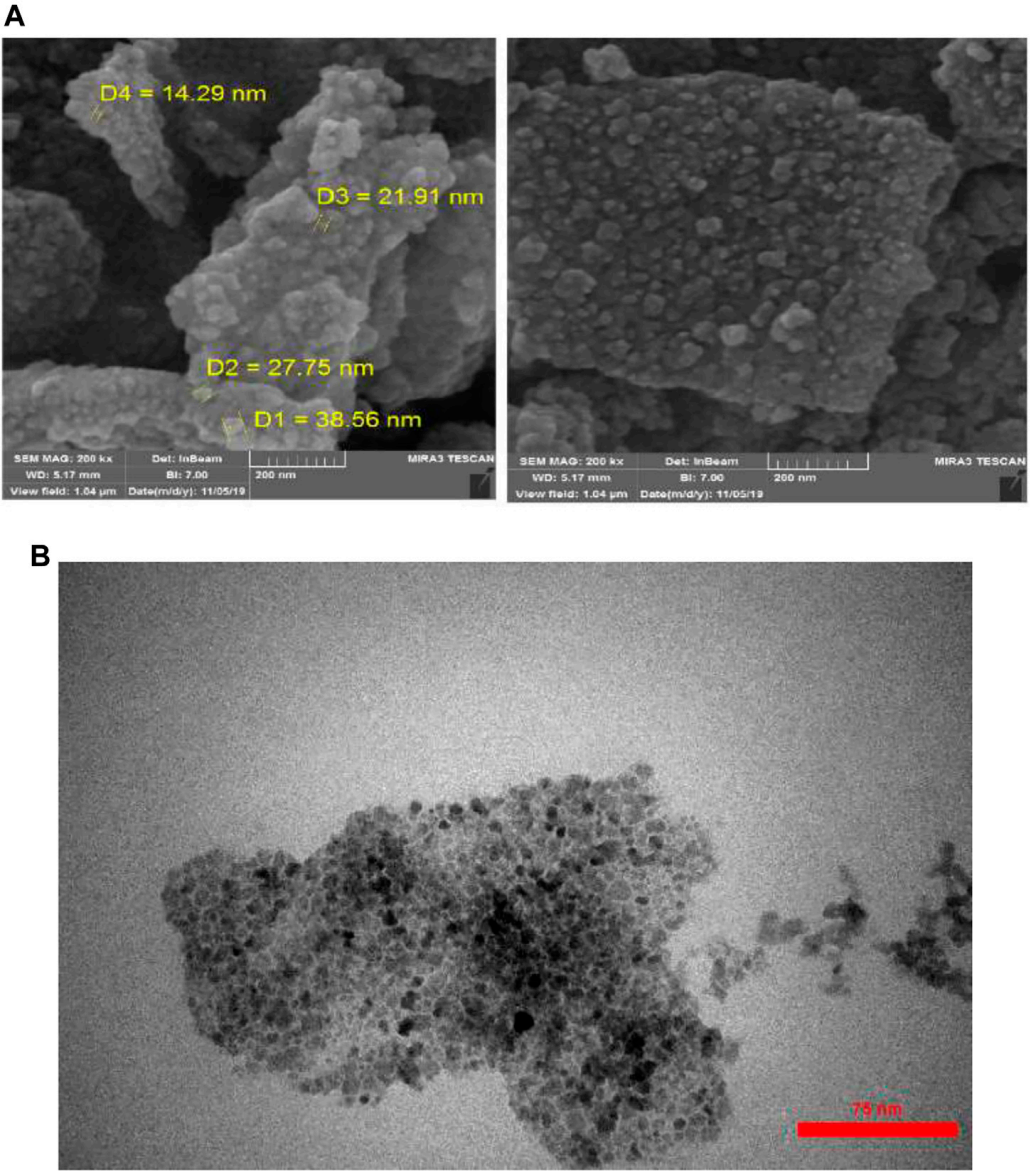
**(A)** FE-SEM and **(B)** TEM images of synthesized Fe_3_O_4_@SiO_2_-pr@dapsone-Cu.

As shown in Figure 1 (FE-SEM and TEM), the magnetic nanoparticles have a spherical shape with an average diameter of 14–38 nm. The synthesized nanoparticles have aggregated well.

**FIGURE 2:**
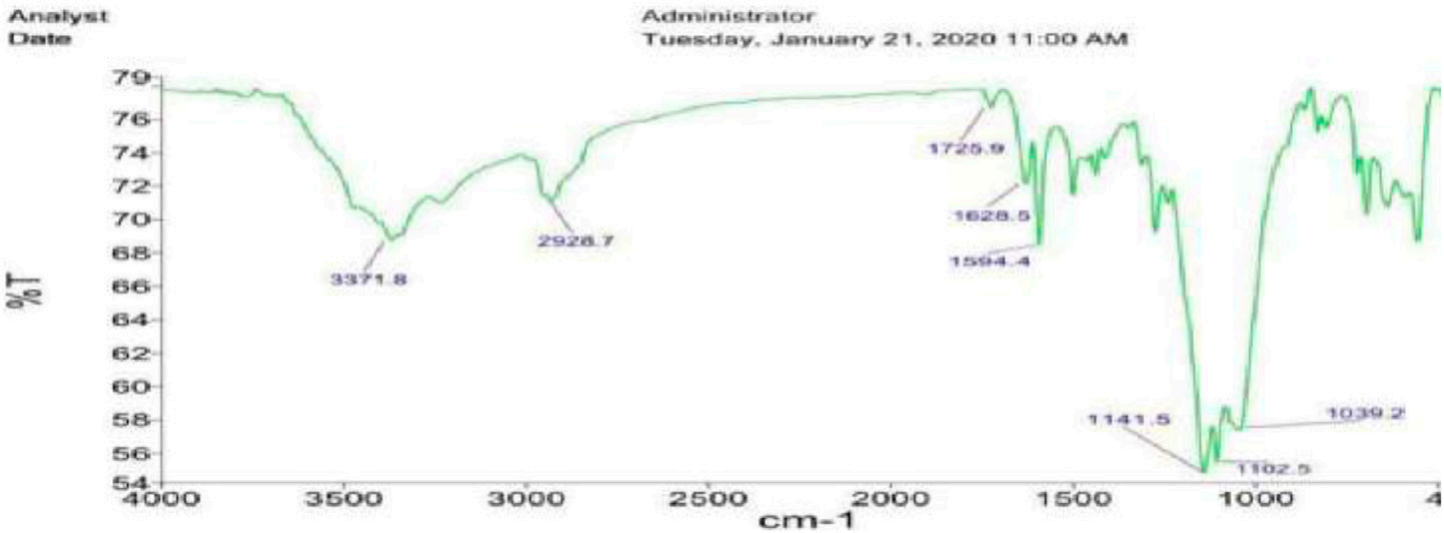
FT-IR spectra of Fe_3_O_4_@SiO_2_-propyl@dapsone-Cu MNPs.

Figure 2 shows FT-IR spectra of Fe_3_O_4_@SiO_2_-propyl@dapsone-Cu MNPs. Wavenumbers of Fe-O bonds of Fe_3_O_4_ appear in 569 and 467 cm^−1^ in Fe_3_O_4_@SiO_2_-propyl@dapsone-Cu MNPs. The peaks positioned at 2,928 cm^−1^ are assigned to aliphatic C-H bonds of Fe_3_O_4_@SiO_2_-propyl@dapsone-Cu MNPs. The peak at 1,102 cm^−1^ and 1,141 cm^−1^ is attributed to Si-O-Si stretching modes of Fe_3_O_4_@SiO_2_-propyl@dapsone-Cu. Furthermore, the other peaks were seen as 1,594 (S=O stretching), 1,628 (aromatic C=C stretching), 3,371 (N-H stretching), and 1,343 cm^−1^ (C-N stretching) in Fe_3_O_4_@SiO_2_-propyl@dapsone-Cu.

**FIGURE 3:**
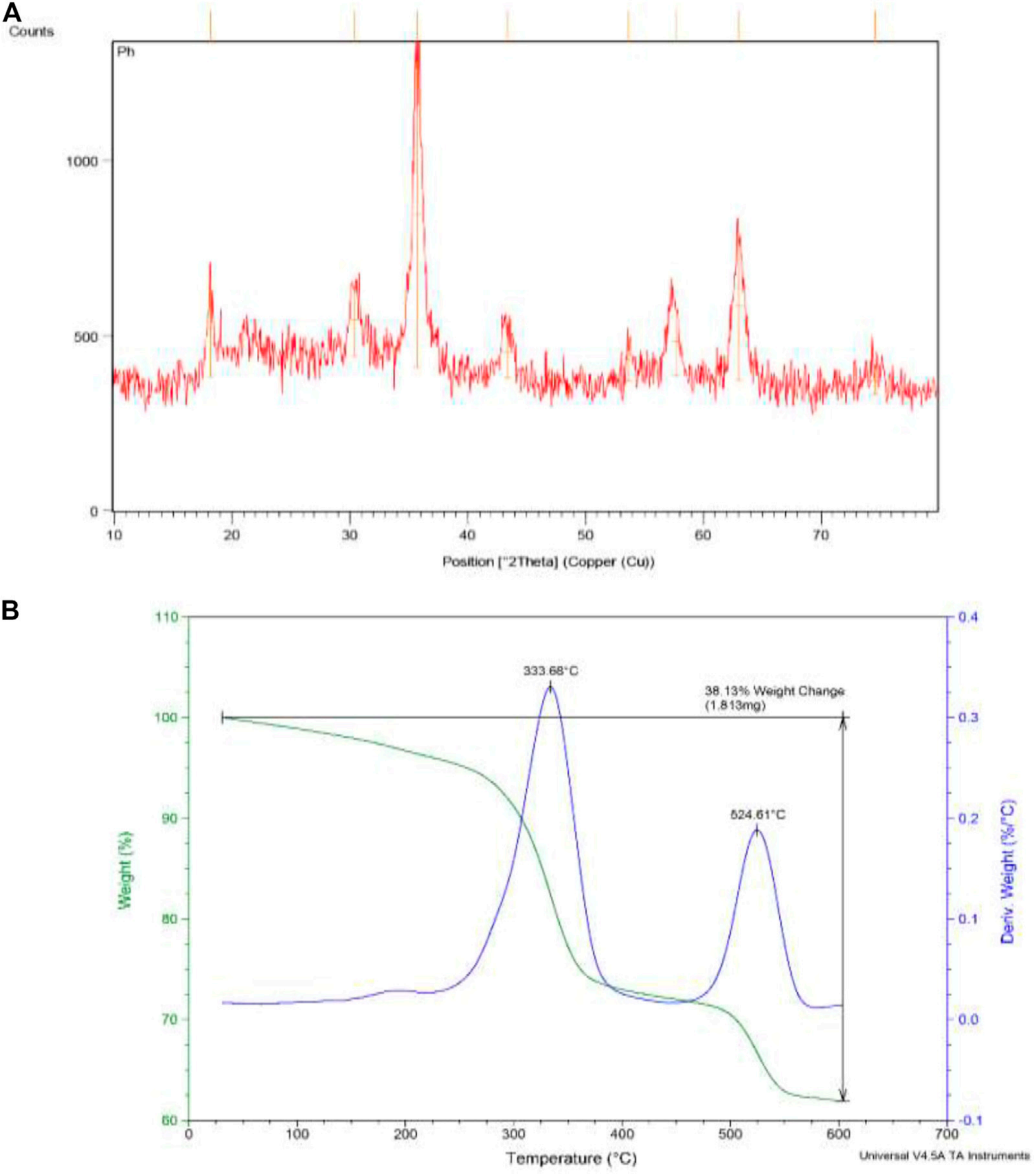
**(A)** The XRD and **(B)** thermogravimetry analysis of synthesized nanoparticles Fe_3_O_4_@SiO_2_-propyl@dapsone-Cu complex.

The XRD pattern of synthesized nanoparticles showed the sharp diffraction peaks with 2θ at 18° (111), 30.4° (220), 35.7° (311), 43.3° (400), 53.6° (422), 57.7° (511), 63.0° (440), and 74.6 (533) (Figure 3A), which indicate that the MNPs have highly crystalline cubic spinel structure of the magnetite and matched with the diffraction patterns of the standard Fe_3_O_4_ (JCPD 19–0629). This confirmed the stability of the crystalline phase of the magnetite core in the structure after silica coating, condensation, and complexation process. The absence of an amorphous peak in pattern confirmed the crystalline structure. Using *Debye–Scherrer equation*, the mean size of crystallite was calculated as 12.1 nm from the XRD pattern (crystallite shape factor: 0.9 and λ _CuKa1_ = 1.54060 Å). This value is lower than the size obtained by FE-SEM and TEM due to the fact that some crystallite forms a particle. Also, the d-spacing and full width at half maximum (FWHM) of the highest XRD peak (2θ = 35.71^o^) were obtained as 2.514 Å and 0.6888 (2θ^o^).

Figure 3B revealed the TGA analysis of synthesized nanoparticles. Two weight losses are observed. The first decrease is related to a temperature below 333°C because of desorption of water and the second weight-loss step at 524°C is due to decomposition of organic compound as dapsone.

**FIGURE 4:**
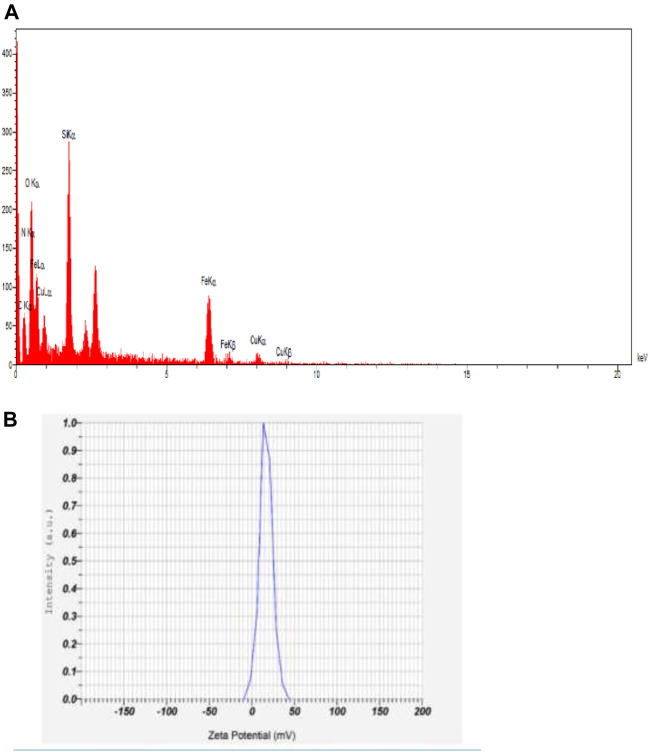
**(A)** Zeta potential and **(B)** EDX analysis of Fe_3_O_4_@SiO_2_-propyl@dapsone-Cu complex.

As shown in Figure 4A, the zeta potential was scanned. The large zeta potential obtained revealed a more stable dispersion of synthesized MNPs. The zeta potential value of dispersed synthesized in deionized water in absence of any electrolyte was +25.1 mV.

The presence of iron, oxygen, nitrogen, carbon, silica, sulfur, and copper, in EDX, revealed the successful synthesis of these nanoparticles.

**FIGURE 5:**
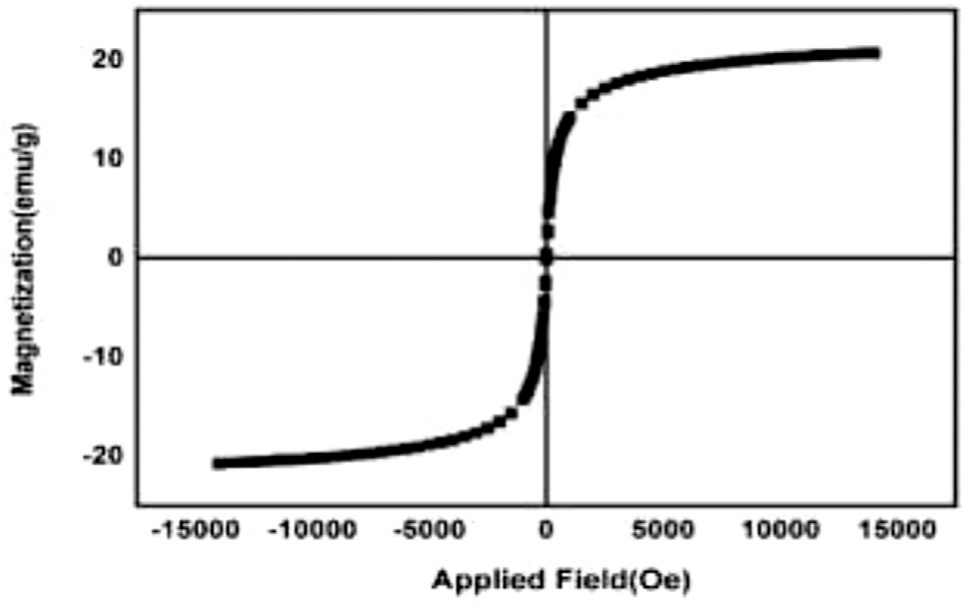
VSM analysis of nanoparticles.

The magnetic properties of synthesized nanoparticles are shown in Figure 5. The results approve the superparamagneticity behavior.

### Catalytic Application

**SCHEME 2:**
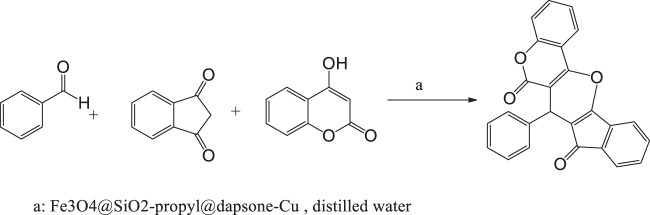
Multicomponent synthesis of pyrano[3,2-c]chromene-diones.

In continuation of our study to synthesize pharmaceutical compounds (Fekri et al., 2018a, b; Fekri and Fard, 2016; Zare et al., 2010; Nikpassand et al., 2012; Fekri and Nikpassand 2014), we triggered to use Fe_3_O_4_@SiO_2_-propyl@dapsone-Cu nanoparticles, for the multicomponent synthesis of novel derivatives of pyrano[3,2-c]chromene-diones via the multicomponent reaction between various aldehydes, indan-1,3-dione, and 4-hydroxycoumarine (Scheme 2).

To complete our assessment, we checked the effect of different conditions in the sample reaction. For example, we treated 4-nitrobenzaldehyde, indandione, and 4-hydroxycoumarin under stirring at room temperature and refluxing in EtOH. The satisfactory results were obtained via the reaction of 4-nitrobenzaldehyde, indandione, and 4-hydroxycoumarin in the presence of 0.05 g of Fe_3_O_4_@SiO_2_-propyl@dapsone-Cu in aqueous media under stirring (Table 1).

**TABLE 1:** The optimization reaction conditions for the synthesis of 4a.

Catalyst	Condition	Time (h)	Yield (%)
—	Refluxing in EtOH	48	11
HCl (concentrated)	Refluxing in EtOH, 10drops	48	28
AcOH	Refluxing in EtOH, 0.1 ml	48	35
K10	Refluxing in EtOH, 0.1 g	24	45
HY-zeolite	Refluxing in EtOH, 0.1 g	24	51
DBU-Ac	Heating at 90°C, 0.5 ml	30	43
DABCO-diAc	Heating at 90°C, 0.5 ml	26	47
Nano-Fe_3_O_4_	Refluxing in EtOH, 0.1 g	12	58
Fe_3_O_4_@SiO_2_	Refluxing in EtOH, 0.1 g	6	62
Fe_3_O_4_@SiO_2_-pr	Refluxing in EtOH, 0.1 g	6	64
Fe_3_O_4_@SiO_2_-Pr@dapsone	Refluxing in EtOH, 0.1 g	5	73
Fe_3_O_4_@SiO_2_-propyl@dapsone-Cu	Refluxing in EtOH, 0.1 g	3	97
Fe_3_O_4_@SiO_2_-propyl@dapsone-Cu	Refluxing in EtOH, 0.01 g	6	76
Fe_3_O_4_@SiO_2_-propyl@dapsone-Cu	Refluxing in EtOH, 0.05 g	3	96
Fe_3_O_4_@SiO_2_-propyl@dapsone-Cu	Stirring at room temperature in water, 0.05 g	3	98

To expand the generality and efficiency of this avenue, some aldehydes with electron-donating or electron-withdrawing substituents were treated with indan-1,3-dione and 4-hydroxycoumarin. The results are summarized in Table 2.

**TABLE 2:** The reaction scope of synthesis of pyrano[3,2-c]chromene-diones.

Entry	Product	Time (h)	Yield (%)^a^ ^,^ ^b^	Mp (°C)
1	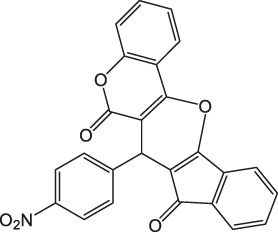	3	98	203–205
2	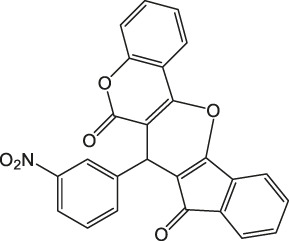	3	96	176–177
3	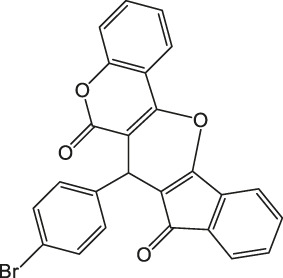	3.5	97	276–278
4	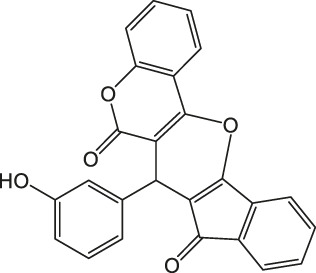	4	94	289–290
5	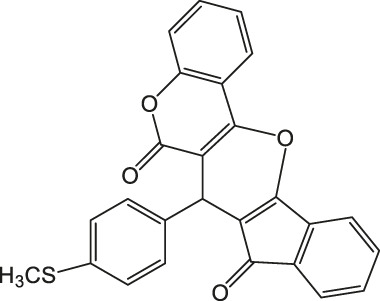	4	95	269–270
6	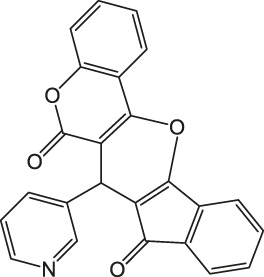	4	96	>300
7	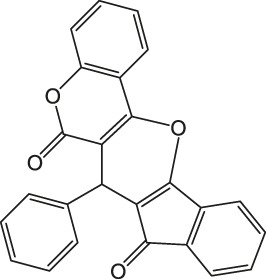	5	94	203–205
8	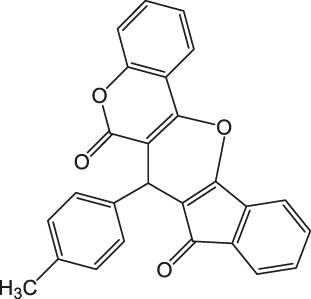	5.5	90	234–235

a Isolated yield.

b All of the synthesized compounds are new and are characterized by FT-IR, NMR, and elemental analysis.

**SCHEME 3:**
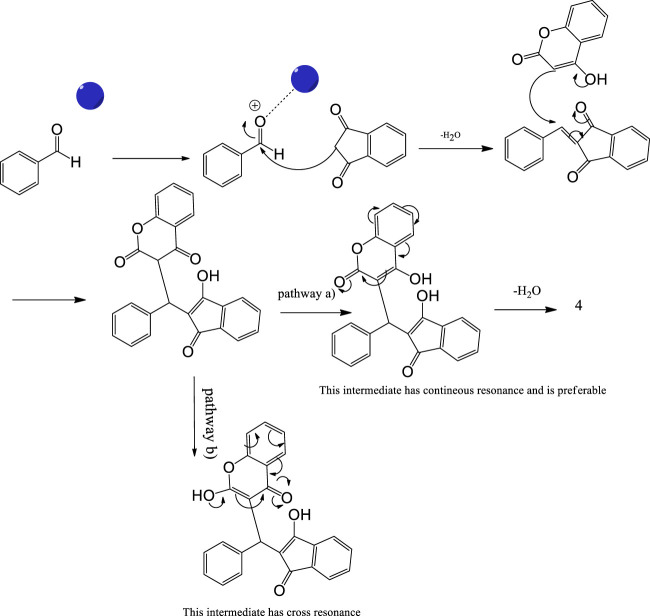
Proposed mechanism for the synthesis of pyranochromene-diones.

As a proposed mechanistic pathway, initially, aldehyde was activated by the nanocatalyst, followed by nucleophilic attack of C-H acid of indan-1,3-diones, together with the departure of water, and chalcone was produced. Nucleophilic attack of 4-hydroxycoumarin to chalcone as Michael addition and then intramolecular cyclization followed by elimination of water lead to product 4 (Scheme 3).

**FIGURE 6:**
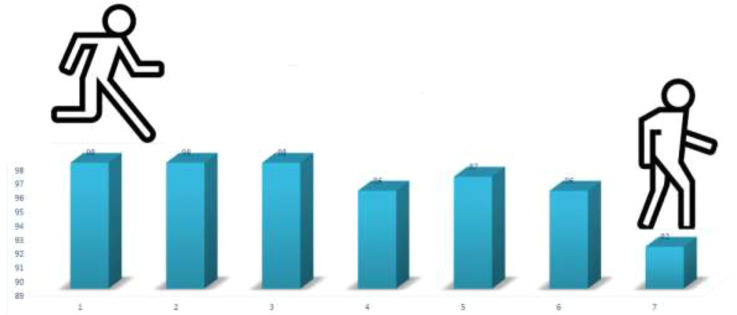
The recyclability of nanocatalyst.

Furthermore, the magnetic nanoparticles are magnetically recoverable and can be reused for six runs. Appearance features of the catalyst were not changed after several uses (Figure 6).

**FIGURE 7:**
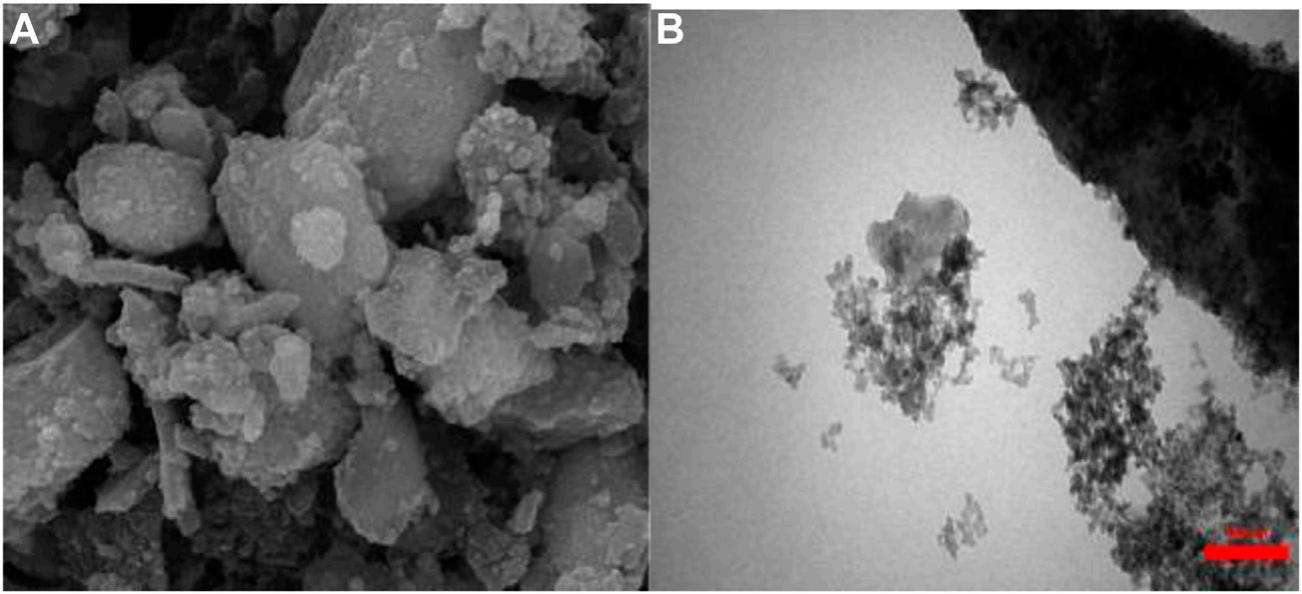
**(A)** FE-SEM and **(B)** TEM image of reused synthesized catalyst in the sixth run.

To better understand the stability of catalyst after five cycles under these reaction conditions, FE-SEM and TEM analyses were carried out. The results are summarized in Figure 7.

## Conclusion

In conclusion, a new catalytic method for the synthesis of pyrano[3,2-c]chromene-diones has been developed. This method offers several advantages, such as simple workup and purification procedure without the use of any chromatographic method, mild reaction conditions, use of inexpensive and commercially available starting materials, recyclability and reusability of the catalyst, high product yields, and short reaction time. So we think that this procedure could be considered a new and useful addition to the present methodologies in this area.

## Data Availability

The original contributions presented in the study are included in the article/supplementary files, further inquiries can be directed to the corresponding author.
